# Quantitative parameters of carotid plaque based on dual-layer spectral-detector CT for stratifying cerebral small vessel disease

**DOI:** 10.3389/fneur.2026.1911566

**Published:** 2026-10-21

**Authors:** Yuxin Ye, Lin Lin, Yuxuan Liu, Chunmiao Lin, Yuyun Xu

**Affiliations:** 1 The Second School of Clinical Medicine, Hangzhou Normal University, Hangzhou, Zhejiang, China; 2 Center for Rehabilitation Medicine, Department of Radiology, Zhejiang Provincial People's Hospital (Affiliated People's Hospital), Hangzhou Medical College, Hangzhou, Zhejiang, China; 3 School of Public Health, Hangzhou Medical College, Hangzhou, Zhejiang, China

**Keywords:** carotid atherosclerosis, carotid non-calcified plaque, cerebral small vessel disease, dual-layer spectral-detector computed tomography, effective atomic number

## Abstract

**Objective:**

Cerebral small vessel disease (CSVD) is a major cause of vascular cognitive impairment and stroke, and has been linked to carotid atherosclerotic plaque characteristics. This study aimed to investigate the association between quantitative parameters of non-calcified carotid plaques derived from dual-layer spectral-detector computed tomography (DLSCT) and CSVD burden, and to construct classification models incorporating clinical and imaging variables.

**Methods:**

This cross-sectional study enrolled 216 patients who underwent carotid DLSCT and brain magnetic resonance imaging. CSVD burden was assessed using a total score (0–4) and categorized as high (score ≥2) or low (score <2) burden. Four logistic regression models were constructed to classify individuals with high CSVD burden, including a clinical model, a model integrating clinical variables with conventional CT features, a model integrating clinical variables with DLSCT-derived parameters, and a model integrating clinical variables, conventional CT features and DLSCT-derived parameters. Model performance was evaluated using optimism-corrected AUC, calibration slope, Brier score, and decision curve analysis.

**Results:**

Age and Z-Zeff were identified as independent correlates, with Z-Zeff demonstrating a particularly notable inverse association (OR = 0.386–0.387, *p* < 0.001). Models incorporating Z-Zeff showed higher discrimination (AUC = 0.748 and 0.759, respectively) compared with the clinical model and the model integrating clinical variables with conventional CT features (AUC = 0.624 and 0.639). Decision curve analysis suggested that models including Z-Zeff had higher net benefit across a range of threshold probabilities.

**Conclusion:**

DLSCT-derived Zeff represents an independent quantitative imaging biomarker associated with CSVD burden. Integrating Z-Zeff with clinical variables was associated with improved discrimination for high CSVD burden, supporting further investigation of DLSCT-derived parameters as imaging correlates of CSVD burden.

## 1 Introduction

Cerebral small vessel disease (CSVD) is one of the leading causes of cognitive dysfunction, ischemic stroke, and dementia in the elderly. Its prevalence increases significantly with age and has become a major global public health concern (1, 2). Early identification of CSVD risk factors and characterization of CSVD burden are important for understanding patient status. Carotid atherosclerosis is closely linked to CSVD progression (3). Previous studies have demonstrated that carotid plaque characteristics correlate with CSVD scores, particularly with the severity of white matter hyperintensities (WMH) (4–6). However, the risk posed by carotid plaques depends not only on their size and stenosis severity, but also on internal plaque composition—including lipid core dimensions, fibrous cap thickness, and the presence of intraplaque hemorrhage (IPH) or necrosis—which collectively determine plaque vulnerability and may be associated with CSVD burden (7, 8). Therefore, accurate assessment of carotid plaque composition may provide new imaging clues for identifying CSVD burden.

Computed tomography angiography (CTA) is currently the primary non-invasive technique for evaluating carotid atherosclerotic plaques (9). Although CTA accurately quantifies luminal stenosis and delineates overall plaque morphology, overlapping tissue attenuation on conventional CT fundamentally impedes the differentiation and quantification of individual plaque components (10). Furthermore, subjective radiological interpretation significantly limits the identification accuracy and reproducibility of plaque assessment.

Dual-layer spectral-detector CT (DLSCT) enables comprehensive multi-parametric imaging in a single acquisition, offering advantages of reduced radiation dose, rapid scanning, and rich post-processing options. Compared to conventional CT attenuation values, DLSCT parameters could better quantify tissue atomic composition and provide superior sensitivity for characterizing non-calcified plaque components—including fibrous tissue, lipid-rich necrotic core (LRNC), and IPH—among which effective atomic number (Zeff) is particularly notable (11–14).

Recent studies have demonstrated that DLSCT parameters may identify carotid plaques associated with ischemic stroke risk (15–18). However, the utility of DLSCT parameters for quantifying cerebral small vessel disease (CSVD) burden remains unexplored. In light of the overlapping vascular pathophysiology linking carotid atherosclerosis and CSVD, quantitative metrics derived from DLSCT may serve as imaging biomarkers associated with CSVD burden.

To explore this hypothesis, this study aimed to characterize associations between DLSCT quantitative parameters of non-calcified carotid plaques and CSVD burden, and to develop exploratory classification models integrating both imaging findings and clinical variables to support identification of individuals with high CSVD burden.

## 2 Materials and methods

### 2.1 Patient selection

This study was conducted in accordance with the Declaration of Helsinki and its subsequent amendments. The Ethics Committee (Approval Number: QT2026005) approved this study, and informed consent was obtained from all participants.

This cross-sectional study prospectively enrolled hospitalized patients who underwent carotid CTA and brain magnetic resonance imaging (MRI) as part of routine evaluation for neurological symptoms suggestive of cerebrovascular pathology. Clinical indications for CTA included suspected carotid disease, recent transient ischemic attack (TIA)/minor stroke, or evaluation of carotid burden in patients with neurological manifestations. MRI indications included evaluation of ischemic/hemorrhagic events, characterization of white matter abnormalities, and comprehensive CSVD marker assessment. Inclusion criteria were: (1) age ≥40 years; (2) CTA confirmed atherosclerotic plaque in at least one carotid artery; (3) concurrent brain MRI [including T1-weighted imaging (T1WI), T2-weighted imaging (T2WI), T2-fluid attenuated inversion recovery imaging (T2-FLAIR), and diffusion-weighted imaging (DWI)/susceptibility-weighted imaging (SWI)] performed within 30 days of the CTA. Exclusion criteria were: (1) history of carotid endarterectomy or stent placement; (2) definite occlusion of the carotid artery or major intracranial arteries (anterior cerebral artery (ACA) or middle cerebral artery (MCA), and vertebrobasilar arteries); (3) maximum carotid atherosclerotic plaque thickness <1.5 mm or calcification > 50%; (4) non-atherosclerotic lesions (dissection, aneurysm, etc.); (5) history of atrial fibrillation; (6) other neurological diseases (Alzheimer's disease, multiple sclerosis, severe brain injury, etc.); (7) acute large-territory infarction or previous large-area old cerebral infarction (diameter > 15 mm), or multiple infarcts/hemorrhages (≥ 3 lesions); (8) poor image quality. A total of 216 patients were enrolled, among whom 10 (4.6%) had acute small infarction, 88 (40.7%) had previous ischemic stroke with old small infarct (maximum diameter ≤ 15 mm) or TIA, and 17 (7.8%) had symptomatic carotid disease. Figure 1 displays the overall study design.

**Figure 1:**
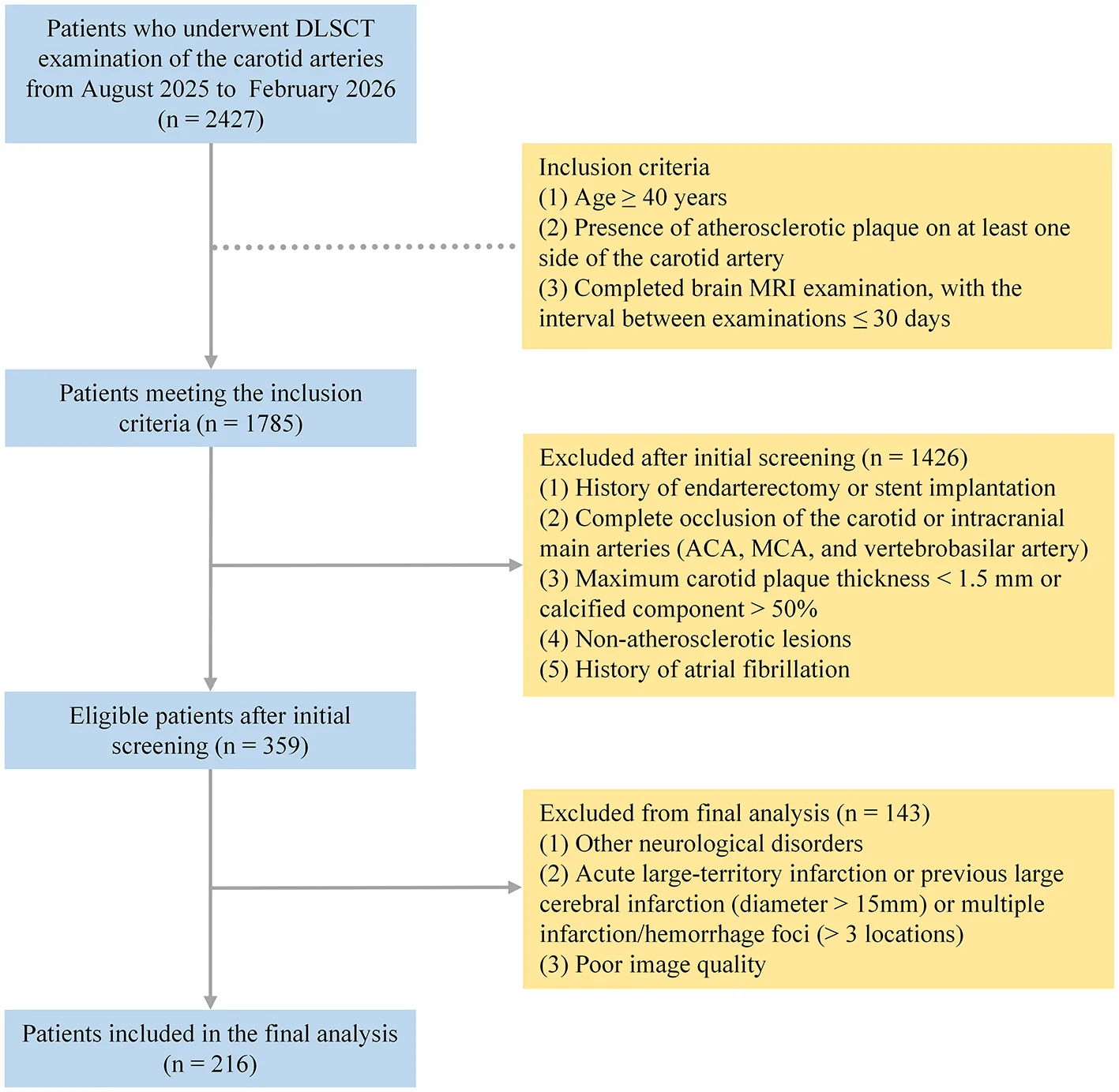
Study flow diagram illustrating patient inclusion and exclusion criteria. DLSCT, dual-layer spectral-detector computed tomography; MRI, magnetic resonance imaging; ACA, anterior cerebral artery; MCA, middle cerebral artery.

### 2.2 Clinical and laboratory data

Demographic information [sex, age, and body mass index (BMI)] and clinical data were retrieved from electronic medical records, including: hypertension (systolic blood pressure ≥140 mmHg, diastolic blood pressure ≥90 mmHg), antihypertensive drug use, hyperlipidemia, statin use, diabetes (or use of hypoglycemic agents), smoking history (current or former), coronary heart disease, antiplatelet drug use (documented regular use at admission), acute small infarction, previous ischemic stroke with old small infarct (maximum diameter ≤ 15 mm) or TIA, and symptomatic carotid disease. Laboratory data included C-reactive protein (CRP), white blood cell (WBC) count, total cholesterol (TC), triglycerides (TG), low-density lipoprotein cholesterol (LDL-C), non-high-density lipoprotein cholesterol (non-HDL-C), remnant cholesterol (defined as non-HDL-C minus LDL-C) (19, 20), homocysteine, and lipoprotein (a) levels.

### 2.3 CTA acquisition protocol

All patients underwent head and neck CT angiography using a Philips Hawk Spectral CT 7,500 scanner (Philips Healthcare, The Netherlands). Patients were placed in the supine position with the head immobilized. The scan range extended from the aortic arch to the vertex. Iohexol (350 mg/ml; 40 ml at 4 ml/s) was administered via a peripheral antecubital venous access using an automated dual-barrel power injector, followed by a 30 mL bolus of isotonic saline. CT scan parameters: arterial phase with spiral acquisition, tube voltage of 120 kVp, and automatic tube current modulation.

### 2.4 CTA image post-processing and assessment

Image analysis was performed by an experienced radiologist using a dedicated workstation (Philips IntelliSpace Portal, Version 12). Reconstruction of carotid axial series was performed from the raw spectral data with a slice thickness of 0.9 mm and a reconstruction interval of 0.5 mm. The carotid axial series included virtual monoenergetic images (VMI) at 40 keV, 70 keV, and 140 keV, as well as effective atomic number (Zeff) maps, electron density (ED) maps, and iodine density (ID) maps. All DLSCT images were reviewed with a fixed window setting (window width, 550 HU; window level, 150 HU).

Two radiologists with 5 and 8 years of experience, respectively, independently evaluated conventional carotid CTA plaque characteristics, including the degree of carotid luminal stenosis, non-calcified plaque thickness, and plaque ulceration, while blinded to brain MRI results and other clinical data. Discrepancies were resolved by consultation with a third radiologist with 18 years of experience. Carotid plaque locations included the common carotid artery, carotid bifurcation, and the proximal segment of the internal carotid artery (cervical segment). When plaque was present in both carotid arteries or multiple plaques were present within the same carotid artery, the plaque at the most stenotic site on the more severely affected side was selected as the representative plaque (18). The most stenotic site was identified through observation in axial, multiplanar reconstruction (MPR), and curved planar reconstruction (CPR) views.

The degree of carotid luminal stenosis was calculated using the North American Symptomatic Carotid Endarterectomy Trial (NASCET) criteria. Carotid stenosis severity was classified as mild (<30%), moderate (30%−69%), and severe (70%−99%). Stenosis severity of ACA or MCA was classified as no stenosis, mild (<50%), moderate (50–69%), and severe (70–99%) (21). Maximum non-calcified plaque thickness was measured on axial images (10, 22). Plaque ulceration was defined as contrast extension beyond the arterial lumen by ≥1 mm (23). Figure 2 displays the narrowest part of the carotid artery and measures plaque thickness.

**Figure 2:**
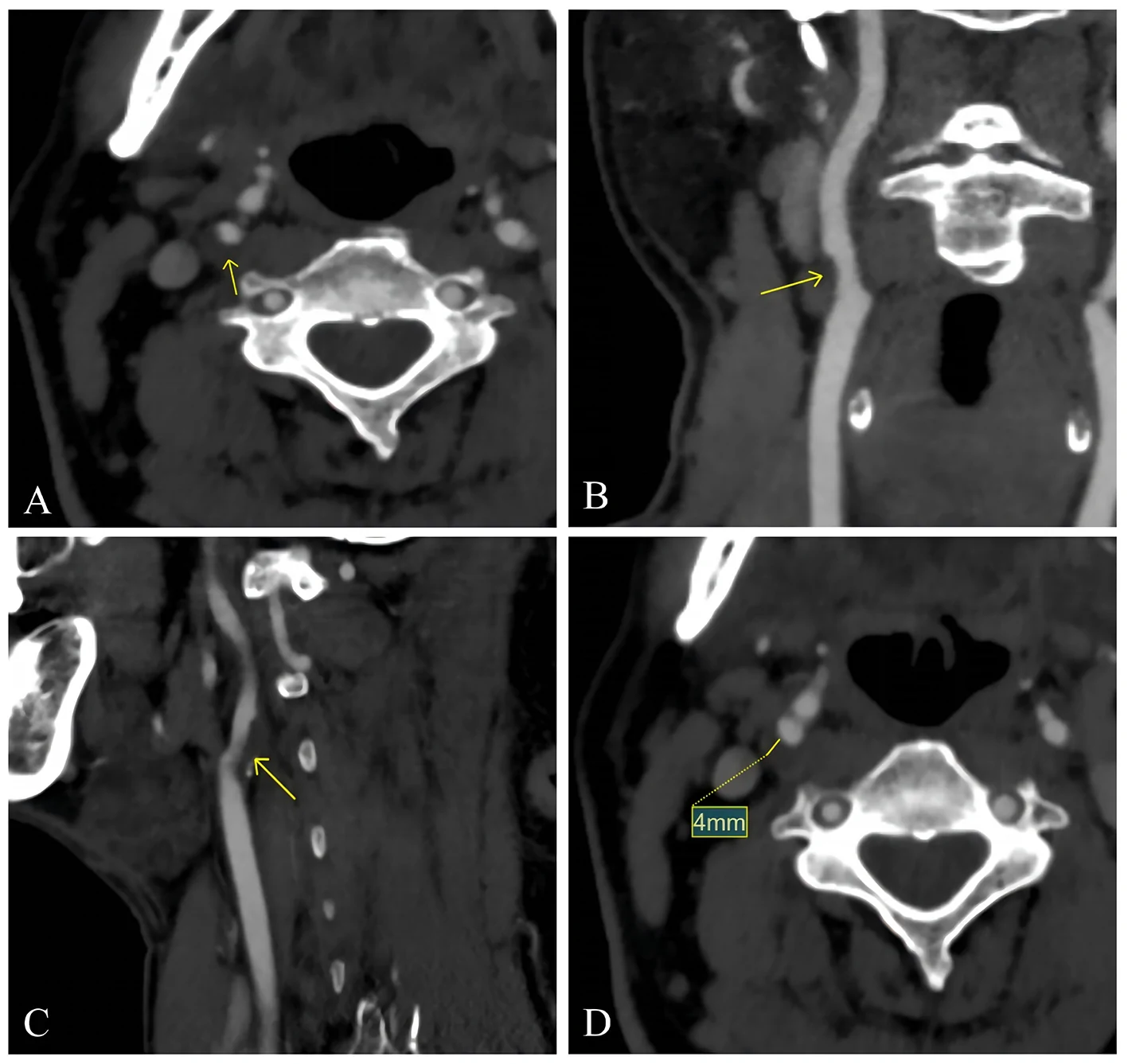
A 50-year-old male patient admitted with dizziness and elevated blood pressure for 6 months. Conventional carotid CTA shows a non-calcified plaque at the right carotid bifurcation with plaque thickness of 4 mm and moderate carotid luminal stenosis. CTA, computed tomography angiography; **(A)** Axial view; **(B)** multiplanar reconstruction (MPR); **(C)** curved planar reconstruction (CPR); **(D)** measurement of non-calcified plaque thickness.

### 2.5 DLSCT parameter assessment

Carotid plaque parameters were independently analyzed by two radiologists with 5 and 8 years of experience using the Philips IntelliSpace Portal workstation. Measurements were performed on the axial slice with the most severe stenosis. A region of interest (ROI) of approximately 1.5–3.0 mm^2^ was placed on the 40 keV VMI, carefully avoiding calcified components. Multiple measurements were obtained within the plaque, and the mean CT values were calculated (24). Once the ROI was placed, it was automatically synchronized to the Zeff map, ED map, ID map, and the 70 keV and 140 keV VMI. CT attenuation values were recorded at each energy level. In addition, the slope of the spectral curve between 40 keV and 140 keV was calculated using the formula (10): λ*HU* = (*HU*140 *keV*−*HU*40 *keV*)/(140 − 40). Fifty cases were randomly selected for repeatability assessment. Two radiologists independently performed blinded evaluations. The first radiologist re-measured the same cases after a 4-week interval to assess intra-rater reliability. Inter-rater and intra-rater reliability were evaluated using the intraclass correlation coefficient (ICC) and Bland-Altman analysis (Supplementary Table S1). Figure 3 displays the assessment of non-calcified carotid plaques using different DLSCT sequences.

**Figure 3:**
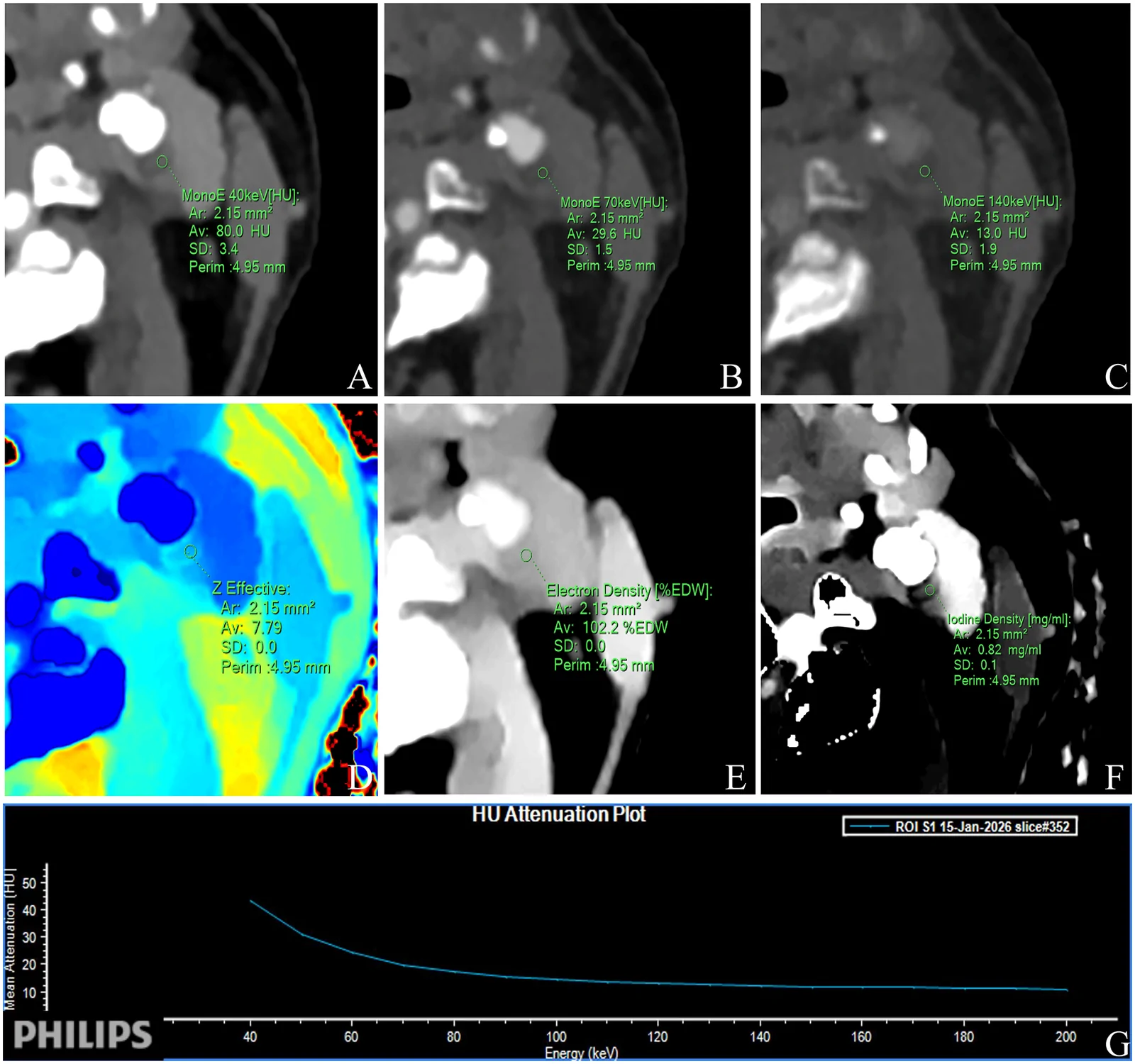
An 82-year-old male patient admitted with slurred speech for 5 years and left lower extremity weakness for 1 day. DLSCT shows a non-calcified plaque at the left common carotid artery bifurcation with moderate carotid luminal stenosis. DLSCT, dual-layer spectral-detector computed tomography; **(A)** VMI at 40 keV; **(B)** VMI at 70 keV; **(C)** VMI at 140 keV; **(D)** effective atomic number (Zeff) map; **(E)** electron density (ED) map; **(F)** iodine density (ID) map; **(G)** spectral curve. VMI, virtual monoenergetic images.

For sensitivity analyses, two alternative Zeff measurements were obtained. Measurable plaques were defined as non-calcified plaques with a thickness ≥1.5 mm on axial images. The lowest Zeff was defined as the lowest Zeff value among all measurable plaques across bilateral carotid arteries. The mean Zeff was defined as the average Zeff value across all measurable plaques. These measurements were performed by the same two radiologists using the same workstation and following the same ROI placement strategy.

### 2.6 MRI acquisition protocol and CSVD marker assessment

MRI examinations were performed using two 3.0-Tesla MRI scanners (Siemens Skyra 3.0 T, Siemens Healthineers, Erlangen, Germany; and GE Discovery MR 750 3.0 T, GE Healthcare, Chicago, IL, USA). Routine MRI sequences included complete T1WI, T2WI, FLAIR, DWI and SWI (Supplementary Table S2). After acquisition, all raw images and reconstructed data were stored in the hospital's Picture Archiving and Communication System (PACS) for subsequent evaluation.

All CSVD markers were defined according to the neuroimaging standards for research into small vessel disease-advances since 2013 (STRIVE-2) (25). White matter hyperintensities (WMH) were defined as hyperintense signals on T2WI and FLAIR sequences, and their severity was quantified using the Fazekas rating scale (grades 0–3) for both periventricular and deep regions (26, 27). Lacunes were defined as round or ovoid, fluid-filled cavities in the subcortical region with 3–15 mm diameter, presenting as central hypointensity with a hyperintense rim on FLAIR. Cerebral microbleeds (CMBs) were defined as small, round hypointense lesions on low-b-value DWI or SWI, typically 2–5 mm in diameter. Perivascular spaces (PVSs) were defined as fluid-filled spaces with cerebrospinal fluid-like signal intensity on all sequences, typically ≤ 3 mm in diameter and classified into three grades based on number of spaces: category 1 (0–10), category 2 (11–25), and category 3 (>25) (25, 28).

Based on these MRI markers, a total CSVD burden score was calculated on an ordinal scale of 0–4 (29–31), with one point assigned for each of the following: (1) ≥1 lacune; (2) deep WMH Fazekas score ≥2 or periventricular WMH Fazekas score ≥3; (3) basal ganglia PVS category ≥2; (4) ≥1 CMB (32). All imaging assessments were performed by two experienced radiologists blinded to clinical information, with consensus obtained from a third senior radiologist in cases of disagreement. Inter-rater and intra-rater agreements for CSVD scoring were evaluated using intraclass correlation coefficients (ICC).

### 2.7 Model construction

Candidate variables were prespecified based on clinical knowledge and previous literature (32–34). The optimal penalty parameter λ was determined using 10-fold cross-validation, and λ. 1se was used to obtain a parsimonious variable set by shrinking coefficients of non-significant variables toward zero. LASSO was used for variable selection and stability assessment, not to define the four models. Age, hypertension, and diabetes were forced into all models as predetermined clinical confounders and were not penalized. Four models for high CSVD burden were established. Model 1 included age, hypertension, and diabetes. Model 2 added non-calcified plaque thickness and carotid lumen stenosis to Model 1. Model 3 added Z-Zeff to Model 1, and Model 4 included all variables simultaneously (age, hypertension, diabetes, non-calcified plaque thickness, carotid lumen stenosis, and Z-Zeff) to determine independently associated factors.

### 2.8 Statistical analysis

Statistical analyses were performed using SPSS (version 27.0, IBM Corp., Armonk, NY, USA) and R software (version 4.5.2, R Foundation for Statistical Computing, Vienna, Austria). The normality of continuous variables was assessed using the Kolmogorov-Smirnov test. Normally distributed data were expressed as mean ± SD and non-normally distributed data were expressed as median (Q1, Q3) and compared using the Mann-Whitney U test. Categorical variables were expressed as *n* (%) and compared using chi-square tests. To eliminate the heterogeneity in measurement units and value ranges, continuous variables entered into the regression models, including age, non-calcified plaque thickness, and DLSCT-derived parameters, were Z-score standardized. The alternative Zeff measurements used in sensitivity analyses (lowest Zeff and mean Zeff) were standardized in the same way. ORs for all standardized variables were expressed per 1-SD increase. To determine whether the ordinal CSVD burden score (0–4) could be analyzed as an ordinal outcome, we performed the Brant test for the proportional odds assumption and attempted to fit a partial proportional odds model. Because the proportional odds assumption was violated and the partial proportional odds model failed to converge, ordinal logistic regression was not considered stable or reliable. The CSVD burden score was therefore dichotomized into high CSVD burden (score ≥2) and low CSVD burden (score <2) for the primary analysis. This binary threshold has been commonly used in prior CSVD studies and has been associated with cognitive decline in large prospective cohort studies (35, 36), supporting it was adopted as the primary outcome definition in the present study. Later, four models were compared using likelihood-ratio tests (α = 0.05) for nested model comparisons. Model discrimination was assessed using the concordance index, with pairwise comparisons of area under the curve (AUC) conducted using a paired DeLong test reporting the difference in AUC (ΔAUC) and 95% confidence intervals. Calibration performance was evaluated using calibration slope and Brier score. Decision curve analysis (DCA) was performed with optimistic bias correction using 1,000 bootstrap resamples. Additionally, the complete variable screening and model-building pipeline was repeated for each bootstrap sample; the AUC, Brier score, and calibration slope for all models after optimism correction were reported. A *p*-value <0.05 was considered statistically significant.

## 3 Results

### 3.1 Patient characteristics and DLSCT parameters

A total of 216 patients were enrolled, with 125 in the low CSVD burden group and 91 in the high CSVD burden group. The distribution of the total CSVD burden score (range 0–4) among the 216 enrolled patients was as follows: Score 0, 46 (21.3%); Score 1, 79 (36.6%); Score 2, 50 (23.1%); Score 3, 35 (16.2%); and Score 4, 6 (2.8%). Patient demographics, conventional CT plaque characteristics, and DLSCT parameters are summarized in Tables 1, 2. The two groups differed significantly in age, smoking status, previous ischemic stroke with old small infarct (maximum diameter ≤ 15 mm) or TIA, statin and antiplatelet drug use (documented regular use at admission), TC, LDL-C, and homocysteine levels (*p* < 0.05), but showed no significant differences in BMI, sex, blood pressure, and blood glucose (*p* > 0.05).

**Table 1:** Comparison of clinical characteristics between high and low CSVD burden groups.

Characteristic	All patients (*n* = 216)	Low CSVD burden (*n* = 125)	High CSVD burden (*n* = 91)	*p*
Age (years)	69.000 (61.000, 75.000)	67.000 (58.000, 74.000)	72.000 (66.000, 76.500)	<0.001
BMI (kg/m^2^)	23.643 ± 3.219	23.540 ± 3.540	23.789 ± 2.730	0.566
Male	147 (68.056)	80 (64.000)	67 (73.626)	0.134
Hypertension	164 (75.926)	89 (71.200)	75 (82.418)	0.057
Systolic pressure (mmHg)	138.032 ± 20.485	137.048 ± 20.146	139.385 ± 20.978	0.409
Diastolic pressure (mmHg)	76.500 (69.000, 85.000)	75.000 (69.000, 85.000)	77.000 (68.000, 84.500)	0.986
Diabetes	83 (38.426)	45 (36.000)	38 (41.758)	0.390
Coronary heart disease	24 (11.111)	12 (9.600)	12 (13.187)	0.408
Smoking	58 (26.852)	26 (20.800)	32 (35.165)	0.019
Acute small infarction	10 (4.630)	4 (3.200)	6 (6.590)	0.241
Previous ischemic stroke with old small infarct (maximum diameter ≤ 15 mm) or TIA	88 (40.740)	24 (19.200)	64 (70.330)	<0.001
Symptomatic carotid artery disease	17 (7.870)	10 (8.000)	7 (7.692)	0.934
Drug				
Antihypertension use	144 (66.667)	77 (61.600)	67 (73.626)	0.064
Statin use	166 (76.852)	89 (71.200)	77 (84.615)	0.021
Antiplatelet use (documented regular use at admission)	141 (65.278)	67 (53.600)	74 (81.319)	<0.001
Laboratory parameters				
CRP (mg/L)	1.300 (0.600, 3.800)	1.300 (0.700, 3.200)	1.400 (0.600, 4.250)	0.769
WBC (× 10^9^/L)	6.040 (5.058, 7.140)	5.850 (5.070, 7.040)	6.240 (5.025, 7.235)	0.179
TC (mmol/L)	3.875 (3.188, 4.630)	4.080 (3.340, 4.670)	3.590 (3.020, 4.360)	0.016
TG (mmol/L)	1.385 (0.907, 1.860)	1.420 (0.920, 1.860)	1.270 (0.895, 1.850)	0.485
Remnant cholesterol (mmol/L)	0.560 (0.420, 0.730)	0.570 (0.450, 0.750)	0.530 (0.415, 0.720)	0.425
LDL-C (mmol/L)	2.346 ± 0.831	2.443 ± 0.814	2.212 ± 0.839	0.043
Non-HDL-C (mmol/L)	2.954 ± 0.975	3.064 ± 0.941	2.803 ± 1.004	0.051
Homocysteine (mmol/L)	12.350 (9.800, 15.700)	11.300 (9.500, 15.100)	12.700 (10.300, 15.750)	0.037
Lp(a) (mg/L)	97.000 (52.000, 248.250)	84.000 (47.000, 200.000)	110.000 (58.000, 304.500)	0.055

Data are presented as mean ± SD, median (IQR), or *n* (%). Antiplatelet use was reported as a baseline treatment-related characteristic and was not included in the final models. CSVD, cerebral small vessel disease; BMI, body mass index CRP, C-reactive protein; WBC, white blood cell; TC, total cholesterol; TG, triglyceride; Non-HDL-C, non-high-density lipoprotein cholesterol; LDL-C, low-density lipoprotein cholesterol; Remnant cholesterol (Non-HDL-C minus LDL-C); Lp(a), lipoprotein(a). SD, standard deviation.

**Table 2:** Comparison of conventional CT and DLSCT parameters between high and low CSVD burden groups.

Variables	All patients (*n* = 216)	Low CSVD burden (*n* = 125)	High CSVD burden (*n* = 91)	*p*
Non-calcified plaque thickness (mm)	2.850 (2.300, 3.625)	2.600 (2.200, 3.400)	3.100 (2.600, 3.800)	0.003
Carotid lumen stenosis				0.061
Mild (<30%)	159 (73.611)	98 (78.400)	61 (67.033)	
≥Moderate (30–99%)	57 (26.389)	27 (21.600)	30 (32.967)	
Plaque ulceration	20 (9.259)	9 (7.200)	11 (12.088)	0.221
HU40KeV	96.100 (63.125, 129.925)	111.300 (83.200, 152.000)	67.800 (48.200, 105.150)	<0.001
HU70KeV	53.142 ± 23.591	59.543 ± 21.286	44.350 ± 23.879	<0.001
HU140KeV	35.369 ± 15.868	39.339 ± 14.182	29.916 ± 16.505	<0.001
λHU	−0.605 (−0.855, −0.350)	−0.719 (−1.091, −0.487)	−0.460 (−0.700, −0.245)	<0.001
Zeff	7.790 ± 0.355	7.897 ± 0.292	7.644 ± 0.382	<0.001
ED	1.034 (1.024, 1.042)	1.036 (1.027, 1.044)	1.029 (1.021, 1.038)	<0.001
ID	0.720 (0.490, 1.143)	0.770 (0.540, 1.160)	0.650 (0.435, 1.130)	0.494

DLSCT, dual-layer spectral-detector computed tomography; CSVD, cerebral small vessel disease; λHU, spectral curve slope; Zeff, effective atomic number; ED, effective electron density; ID, iodine density.

Compared with the low CSVD burden group, the high CSVD burden group had significantly lower HU40 keV, HU70 keV, HU140 keV, λHU, Zeff, and ED (all *p* < 0.05), but significantly higher non-calcified plaque thickness (*p* < 0.05). Iodine density (ID) demonstrated no significant between-group difference (*p* > 0.05). Figure 4 displays the distribution of Zeff, HU40 keV, Z-ED, and Z-λHU across CSVD burden groups.

**Figure 4:**
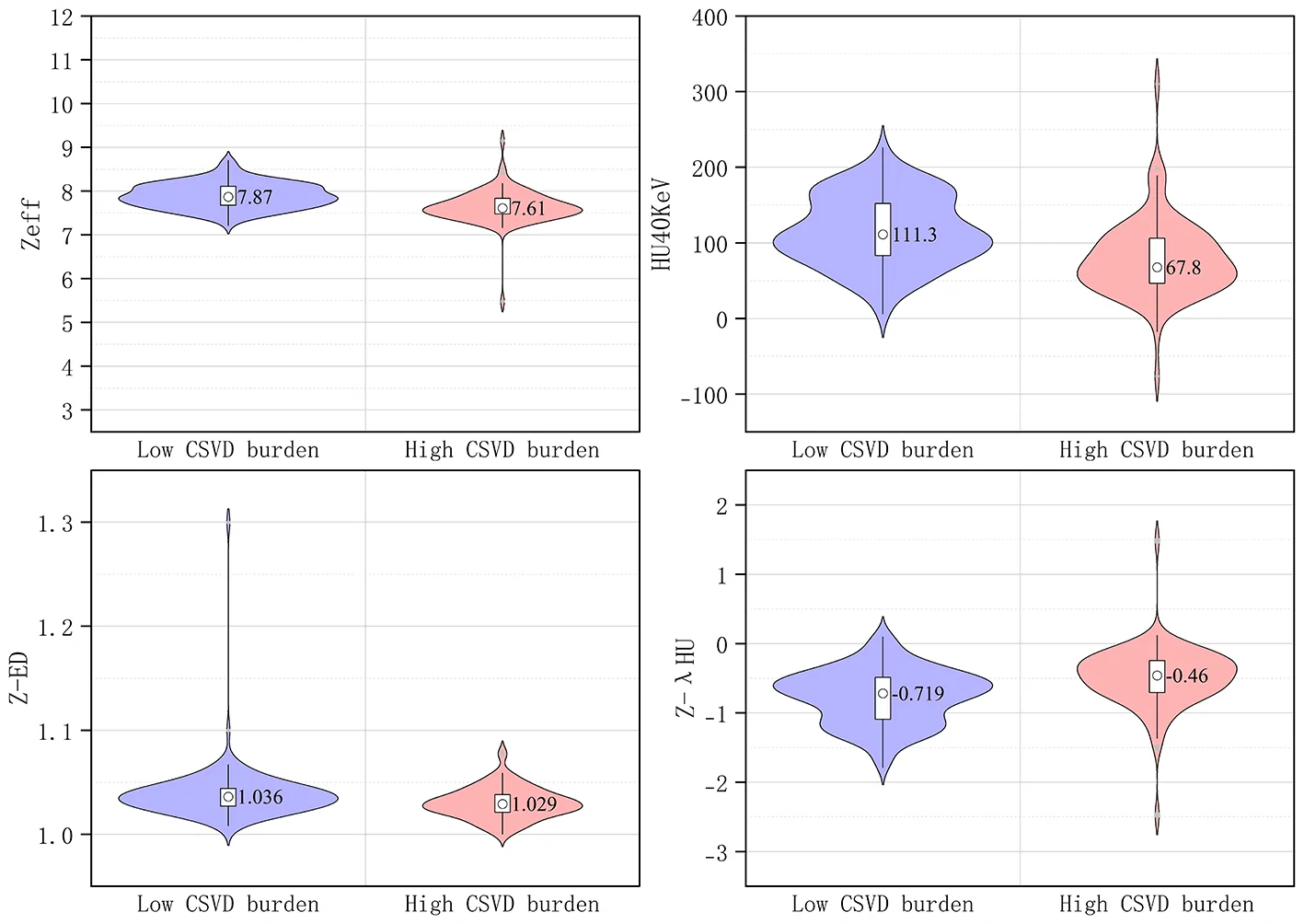
Violin plots showing the distribution of Zeff, HU40 keV, Z-ED, and Z-λHU across CSVD burden groups. Z-score standardized variables are indicated by Z- prefix. CSVD, cerebral small vessel disease; λHU, spectral curve slope; Zeff, effective atomic number; ED, electron density.

### 3.2 Multivariable logistic regression analysis

In variable-specific Brant tests, the proportional odds assumption was violated for Z-Zeff in Models 3 and 4, both *p* < 0.001, and for non-calcified plaque thickness in Model 4 (*p* = 0.020) (Supplementary Table S3). The partial proportional odds model failed to converge, likely because only 35 patients had a CSVD score of 3 and 6 had a score of 4. Therefore, the binary CSVD burden outcome was used for all subsequent analyses.

Multivariable logistic regression analyses for the four models are presented in Table 3. Age was independently associated with higher CSVD burden across all models (*p* < 0.001), with odds ratios (ORs) ranging from 1.776 (95% CI: 1.305–2.416) in Model 1 to 1.922 (95% CI: 1.355–2.728) in Model 4, per 1-SD increase. For the carotid morphological parameters, neither non-calcified plaque thickness nor carotid lumen stenosis (moderate or severe) exhibited significant associations with higher CSVD burden when added in Models 2 and 4 (all *p* > 0.05). In contrast, Z-Zeff showed a significant independent inverse association with higher CSVD burden (per 1-SD increase), remaining highly significant in both Model 3 (OR = 0.387; 95% CI: 0.262–0.572; *p* < 0.001) and Model 4 (OR = 0.386; 95% CI: 0.256–0.581; *p* < 0.001). Figure 5 displays a representative illustration of Zeff values between high and low CSVD burden.

**Table 3:** Multivariable logistic regression analysis results.

Model	Model 1		Model 2		Model 3		Model 4	
Variable	OR (95% CI)	*p*	OR (95% CI)	*p*	OR (95% CI)	*p*	OR (95% CI)	*p*
Z-age	1.776 (1.305–2.416)	<0.001	1.738 (1.260–2.398)	<0.001	1.872 (1.340–2.616)	<0.001	1.922 (1.355–2.728)	<0.001
Hypertension	1.964 (0.978–3.944)	0.058	1.944 (0.953–3.968)	0.068	1.731 (0.816–3.671)	0.153	1.658 (0.770–3.570)	0.196
Diabetes	1.006 (0.557–1.817)	0.984	0.969 (0.533–1.760)	0.917	0.954 (0.507–1.796)	0.883	0.919 (0.486–1.738)	0.795
Z-non-calcified plaque thickness	—		1.316 (0.901–1.923)	0.155	—		1.257 (0.834–1.894)	0.274
Carotid lumen stenosis Moderate (30–69%)	—		0.840 (0.273–2.587)	0.762	—		0.552 (0.168–1.819)	0.329
Carotid lumen stenosis severe (70%−99%)	—		1.144 (0.459–2.851)	0.772	—		0.771 (0.287–2.066)	0.605
Z-Zeff	—		—		0.387 (0.262–0.572)	<0.001	0.386 (0.256–0.581)	<0.001

—: Indicates that this variable is not included in the model. Model 1 = age+hypertension+diabetes; Model 2 = Model 1 +non-calcified plaque thickness+carotid lumen stenosis; Model 3 = Model 1+Z-Zeff; Model 4 = age+hypertension+diabetes+non-calcified plaque thickness+carotid lumen stenosis+Z-Zeff. Hypertension and diabetes were dichotomized, with absence of the condition as the reference category. For carotid lumen stenosis, mild stenosis (<30%) was the reference category, and moderate (30–69%) and severe (70–99%) stenosis were compared with it. All continuous variables (age, non-calcified plaque thickness, and Zeff) were standardized; thus, all OR values represent the change in odds of high CSVD burden per 1-SD increase in the respective standardized variable. OR, odds ratio; CI, confidence interval; Zeff, effective atomic number; SD, standard deviation.

**Figure 5:**
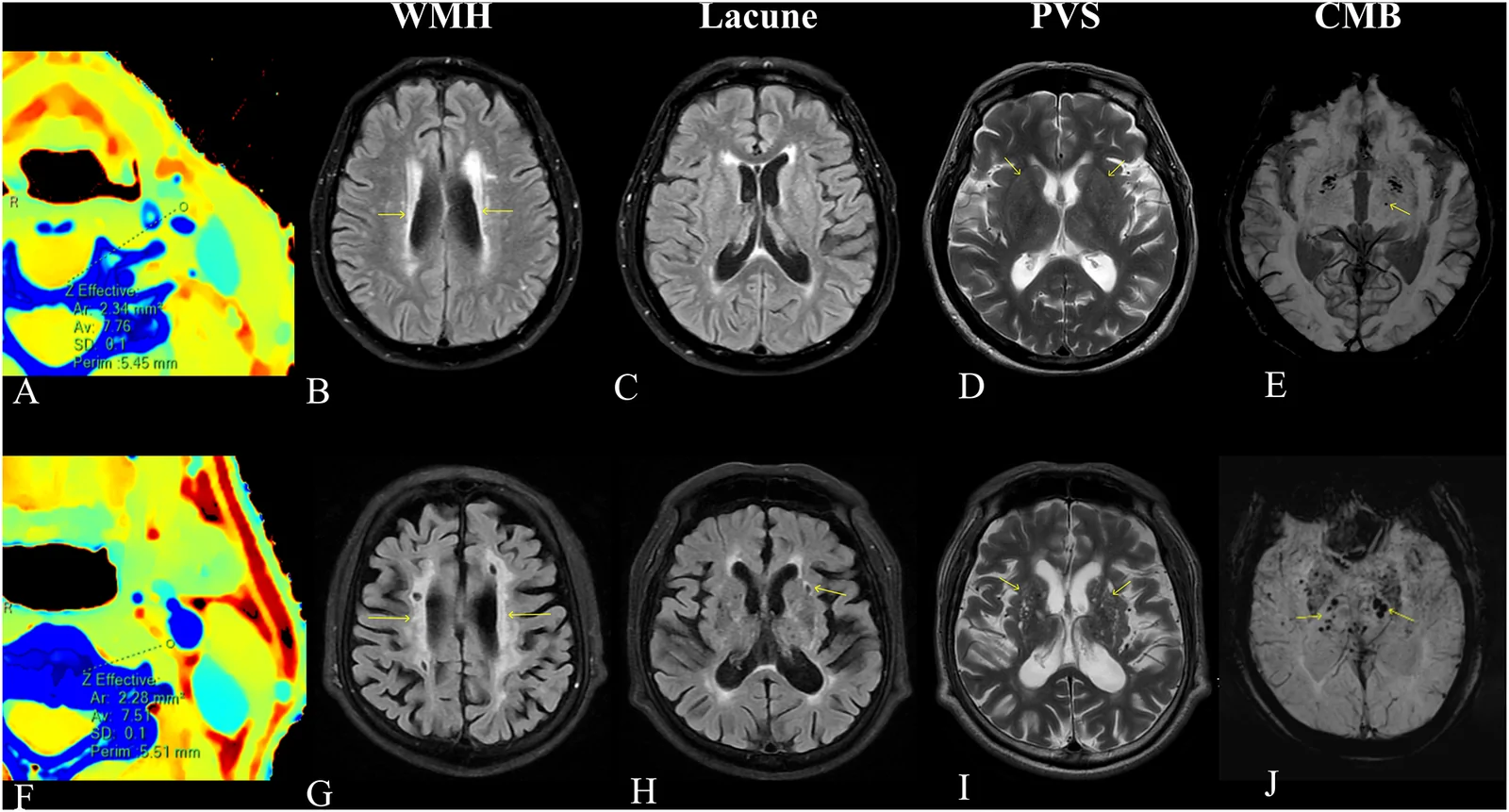
**(A–E)** A 76-year-old male patient admitted with paroxysmal right upper extremity numbness and weakness for 4 h. **(A)** Non-calcified plaque at the left common carotid artery with DLSCT Zeff value of 7.76; brain MRI shows CSVD score of 1. **(B)** Periventricular WMH on FLAIR, Fazekas grade 2. **(C)** No lacune in the basal ganglia on FLAIR. **(D)** Basal ganglia PVS on T2WI, grade 1. **(E)** CMB in the right thalamus on SWI. **(F–J)** A 74-year-old male patient admitted with gait instability and bilateral lower extremity weakness for more than 10 months. **(F)** Non-calcified plaque at the left carotid bifurcation with DLSCT Zeff value of 7.51; brain MRI shows CSVD score of 4. **(G)** Periventricular WMH on FLAIR, Fazekas grade 3. **(H)** Lacune in the left basal ganglia on FLAIR. **(I)** Basal ganglia PVS on T2WI, grade 2. **(J)** Multiple scattered CMBs on SWI. DLSCT, dual-layer spectral-detector computed tomography; Zeff, effective atomic number; MRI, magnetic resonance imaging; CSVD, cerebral small vessel disease; WMH, white matter hyperintensities; FLAIR, fluid attenuated inversion recovery imaging; PVS, perivascular space; T2WI, T2-weighted imaging; CMB, cerebral microbleed; SWI, susceptibility-weighted imaging.

In sensitivity analyses using alternative Zeff measurements, both the lowest Zeff (OR = 0.210, 95% CI: 0.120–0.370, *p* < 0.001) and the mean Zeff (OR = 0.230, 95% CI: 0.140–0.380, *p* < 0.001) remained independently associated with high CSVD burden after adjustment for age, hypertension, diabetes, non-calcified plaque thickness, and carotid lumen stenosis. These results were consistent with the primary analysis using Zeff at the most stenotic plaque (Supplementary Table S4).

### 3.3 Model performance and calibration

The optimism-corrected discrimination and calibration performance of the four models is summarized in Table 4. Models incorporating Z-Zeff (Models 3 and 4) demonstrated significantly better discrimination compared to those without it (Models 1 and 2). The optimism-corrected AUC increased from 0.624 (95% CI = 0.590–0.733) for Model 1 and 0.639 (95% CI = 0.585–0.729) for Model 2, to 0.748 (95% CI = 0.712–0.837) for Model 3 and 0.759 (95% CI = 0.708–0.835) for the fully adjusted Model 4. Regarding calibration, the fully adjusted Model 4 exhibited the best goodness-of-fit, as evidenced by the lowest Brier score (0.199) and a calibration slope of 0.933, which was closest to the ideal value of 1.0. In comparison, Model 1 showed notable miscalibration with a slope of 0.794, indicating some deviation from ideal calibration, while Models 2 and 3 demonstrated moderate calibration slopes of 0.916 and 0.860, respectively. The ΔAUC between Model 4 and Model 2 was 0.120 (95% CI: 0.058–0.183); the DeLong's test *p* < 0.001, indicating a statistically significant difference. Additionally, by conducting a likelihood ratio test to compare the nested models (Model 2 vs. Model 4), we obtained χ^2^ = 27.63, *p* < 0.001, which further supports the significant improvement in model fit after incorporating the Z-Zeff variables (Supplementary Table S5). Collectively, these findings suggest that incorporating Z-Zeff into clinical and conventional imaging factors was associated with improved model discrimination and calibration for identifying higher CSVD burden. Figure 6 shows ROC curves of four CSVD models.

**Table 4:** Discrimination and calibration of the four models for high CSVD burden.

Model	Optimism-corrected AUC	Optimism-corrected AUC 95% CI	Optimism-corrected Brier score	Optimism-corrected calibration slope
Model1	0.624	0.590–0.733	0.236	0.794
Model2	0.639	0.585–0.729	0.233	0.916
Model3	0.748	0.712–0.837	0.204	0.860
Model4	0.759	0.708–0.835	0.199	0.933

Model 1=age+hypertension+diabetes; Model 2=Model 1+non-calcified plaque thickness+carotid lumen stenosis; Model 3=Model 1+Z-Zeff; Model 4=age+hypertension+diabetes+non-calcified plaque thickness+carotid lumen stenosis+Z-Zeff. CSVD, cerebral small vessel disease; AUC, area under the curve; CI, confidence interval; Zeff, effective atomic number; Z-Zeff, Z-score-standardized effective atomic number.

**Figure 6:**
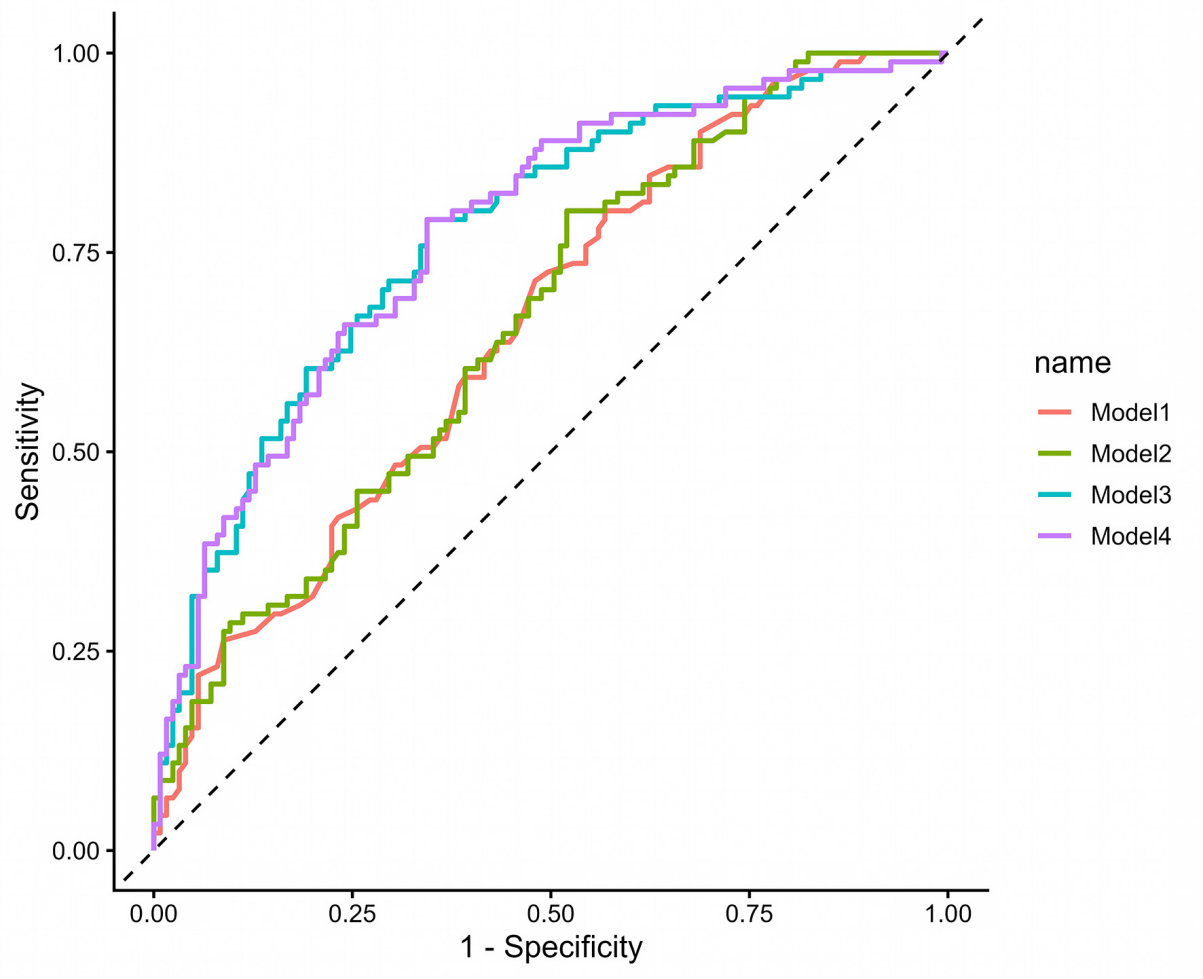
ROC curves of the four models. ROC, receiver operating characteristic. Orange, green, blue and purple correspond to Model 1, Model 2, Model 3, and Model 4, respectively. Model 1 = age+hypertension+diabetes; Model 2 = Model 1 +non-calcified plaque thickness+carotid lumen stenosis; Model 3 = Model 1+Z-Zeff; Model 4 = age+hypertension+diabetes+non-calcified plaque thickness+carotid lumen stenosis+Z-Zeff. Zeff, effective atomic number; Z-Zeff, Z-score-standardized effective atomic number.

### 3.4 Model performance and decision curve analysis

Decision curve analysis with bootstrap optimism correction was performed to evaluate the net benefit of the four models across varying threshold probabilities (Figure 7). Models 3 and 4, which incorporated Z-Zeff, consistently yielded higher net benefits compared with Models 1 and 2 across most threshold ranges. The greatest advantage of Model 4 over the other models was observed at threshold probabilities of 20%−50%, particularly between 30% and 40%. These exploratory findings suggest that Model 4 showed higher net benefit across the evaluated threshold ranges.

**Figure 7:**
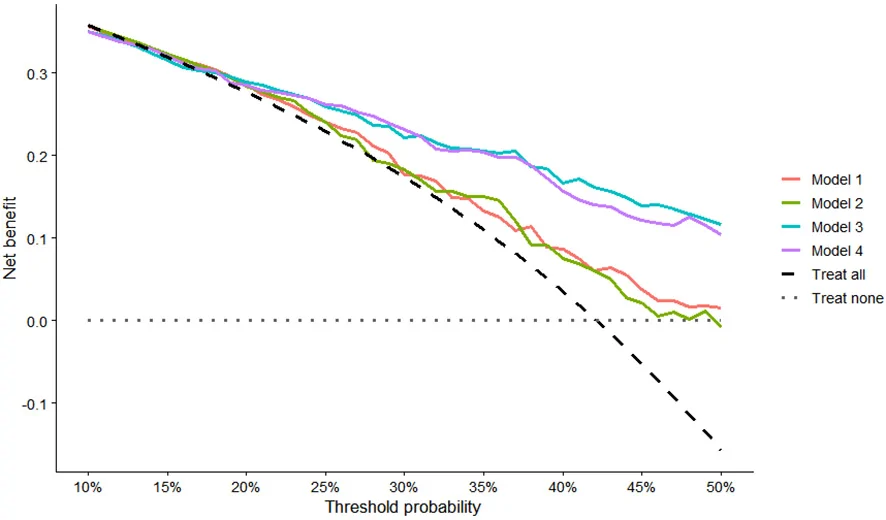
DCA curves after Bootstrap correction of the four models. DCA, decision curve analysis. The y-axis denotes net benefit, while the x-axis indicates threshold probability. Orange, green, blue and purple correspond to Model 1, Model 2, Model 3, and Model 4, respectively. Model 1 = age+hypertension+diabetes; Model 2 = Model 1 +non-calcified plaque thickness+carotid lumen stenosis; Model 3 = Model 1+Z-Zeff; Model 4 = age+hypertension+diabetes +non-calcified plaque thickness+carotid lumen stenosis+Z-Zeff. CSVD, cerebral small vessel disease; Zeff, effective atomic number; Z-Zeff, Z-score-standardized effective atomic number.

### 3.5 Incremental analysis of intracranial large vessels

To determine whether the degree of intracranial artery stenosis (ACA/MCA and vertebrobasilar arteries) has additional stratification value beyond the DLSCT parameters and clinical variables, we conducted univariate and multivariate logistic regression analyses of the stenosis degree as an additional factor (Table 5). The univariate analysis showed that mild and severe ACA/MCA stenosis as well as severe vertebrobasilar artery stenosis were significantly associated with a higher CSVD burden. However, after adjusting for age, the use of antiplatelet drug (documented regular use at admission), and DLSCT parameters, this association no longer had statistical significance in the multivariate analysis (all *p* > 0.05).

**Table 5:** Univariate and multivariate logistic regression analysis of ACA/MCA and vertebrobasilar stenosis associated with CSVD.

Variables	Univariate logistic regression		Multivariate logistic regression	
	OR (95% CI)	*p*	*OR* (95% CI)	*p*
ACA or MCA stenosis				
No stenosis	1.000 (Reference)		1.000 (Reference)	
Mild (<50%)	5.347 (1.532–18.663)	0.009	1.879 (0.433–8.164)	0.4
Moderate (50–69%)	5.333 (0.891–31.918)	0.067	1.776 (0.230–13.697)	0.582
Severe (70–99%)	11.667 (2.075–65.595)	0.005	3.613 (0.483–27.017)	0.211
Vertebrobasilar stenosis				
No stenosis	1.000 (Reference)		1.000 (Reference)	
Mild (<50%)	1.534 (0.789–2.984)	0.207	0.905 (0.373–2.197)	0.826
Moderate (50–69%)	1.154 (0.433–3.073)	0.775	0.838 (0.247–2.842)	0.776
Severe (70–99%)	2.368 (1.131–4.960)	0.022	0.728 (0.282–1.877)	0.511
Z–age	1.770 (1.300–2.420)	<0.001	1.710 (1.130–2.560)	0.010
Antiplatelet use (documented regular use at admission)	3.768 (2.000–7.101)	<0.001	2.611 (1.182–5.767)	0.018
Z-Zeff	0.180 (0.107–0.302)	<0.001	0.199 (0.117–0.337)	<0.001

Z-Age and Z-Zeff indicate Z-score-standardized age and effective atomic number; ORs represent the effect per 1-SD increase. Antiplatelet use was included as a treatment-related covariate and should not be interpreted as an independent causal factor. ACA, anterior cerebral artery; MCA, middle cerebral artery; CSVD, cerebral small vessel disease; OR, odds ratio; CI, confidence interval; Zeff, effective atomic number; SD, standard deviation.

### 3.6 Clinical subgroup stratification and interaction analysis

To assess whether clinical factors modify the association between Z-Zeff and high CSVD burden, we performed subgroup stratification analyses across multiple patient characteristics: age, sex, BMI, smoking status, hypertension, diabetes, hyperlipidemia, coronary heart disease, carotid stenosis degree, and antiplatelet drug use (documented regular use at admission) (Figure 8). This analysis also included a subgroup analysis of the history of stroke/TIA, acute small infarction, and symptomatic carotid disease (Supplementary Table S6). Interaction analysis revealed no statistically significant interactions between Z-Zeff and any clinical factor examined (age: *p* = 0.738; BMI: *p* = 0.602; sex: *p* = 0.761; smoking status: *p* = 0.069; hypertension: *p* = 0.558; diabetes: *p* = 0.188; coronary heart disease: *p* = 0.419; carotid artery stenosis degree: *p* = 0.278; antiplatelet drug use (documented regular use at admission): *p* = 0.186; hyperlipidemia: *p* = 0.498; previous ischemic stroke with old small infarct (maximum diameter ≤ 15 mm) or TIA: *p* = 0.682; acute small infarction: *p* = 0.547; symptomatic carotid disease: *p* = 0.891). These results indicate that the inverse association between Z-Zeff and high CSVD burden was consistent across all examined subgroups, including patients with previous ischemic stroke with old small infarct (maximum diameter ≤ 15 mm) or TIA, acute small infarction, and symptomatic carotid disease.

**Figure 8:**
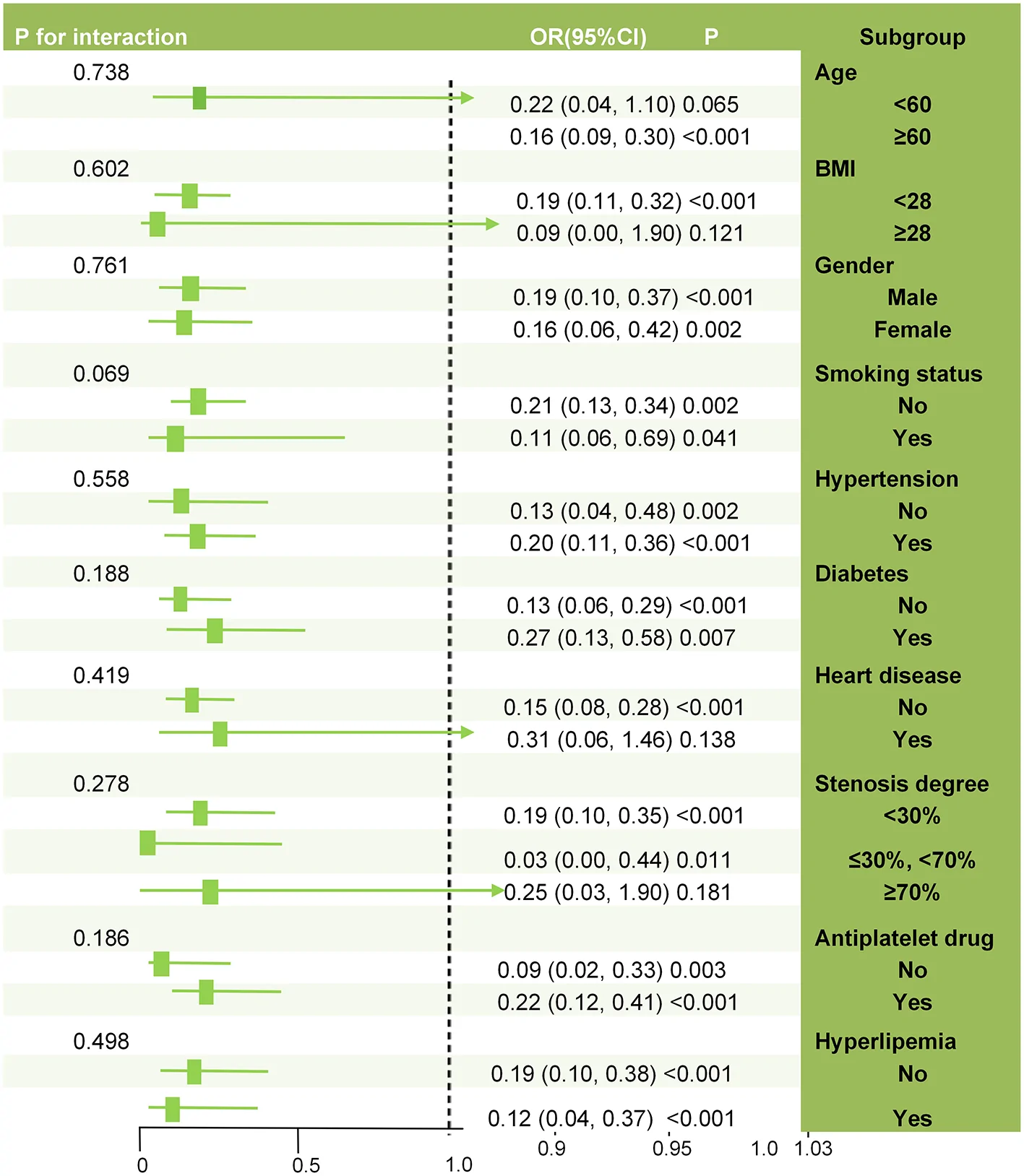
Subgroup analysis of the association between Z-Zeff and high CSVD burden: evaluation of effect modification by clinical factors. BMI, body mass index; CHD, coronary heart disease; Zeff, effective atomic number; Z-Zeff, Z-score-standardized effective atomic number. CSVD, cerebral small vessel disease.

## 4 Discussion

In this study, DLSCT-derived non-calcified carotid plaque parameters were found to be significantly associated with CSVD burden. Based on these findings, we developed a clinical-imaging risk classification model that integrates plaque composition and morphological features with clinical variables. We then evaluated the discriminative performance of this combined model for classifying high CSVD burden. Multivariable logistic regression further identified age and Z-Zeff as independent associated factors, with Z-Zeff demonstrating a particularly robust association with high CSVD burden.

Zeff was derived from quantitative dual-layer spectral-detector CT measures and represents an imaging-based parameter related to plaque composition and activity. Clinically, lower Zeff values may be associated with higher proportions of lipid-rich, hemorrhagic, or necrotic core components—features indicative of plaque vulnerability, whereas higher Zeff values reflect relatively greater fibrous tissue composition, suggesting plaque stability (13, 14, 37). This finding is consistent with its known ability to quantitatively assess plaque components with high sensitivity, which is superior to traditional CT (13, 37). Hua et al. demonstrated the accuracy and repeatability of Zeff derived from DLSCT through phantom measurements (38). By capturing these compositional distinctions, Zeff serves as an objective quantitative imaging biomarker associated with the extent of cerebral small vessel damage, while the precise quantification of plaques by DLSCT overcomes the limitations of conventional CTA, providing additional information for CSVD burden assessment.

Interestingly, ID showed no significant difference between CSVD burden groups (*p* > 0.05), reflecting differences between compositional and perfusion-based biomarkers (37). Zeff quantifies intrinsic tissue composition independent of perfusion, whereas ID is heavily influenced by microvascular perfusion and susceptible to acquisition artifacts. Additionally, ID measurements are particularly sensitive to acquisition timing and individual hemodynamic factors, introducing technical noise that obscures disease-related signals (39). Therefore, the DLSCT-derived compositional biomarker Zeff is more robust than the perfusion-based metric ID, making it more suitable for CSVD assessment.

The observed association between Zeff and CSVD burden may be underpinned by plausible biological processes. We therefore propose three potential (hypothesis-generating) pathways: (1) Systemic inflammation and blood-brain barrier (BBB) disruption: an inflammatory microenvironment within the plaque may drive cytokine release that damages cerebral microvascular endothelium and increases blood-brain barrier permeability, potentially favoring WMH progression and demyelination (40–42). (2) Microembolism: plaques with lower Zeff values tend to show features of instability, and embolic debris arising from such plaques could obstruct distal arterioles, causing focal perfusion disturbances that manifest as WMH, lacunar infarction, enlarged perivascular spaces, and cerebral microbleeds (43–47). (3) Endothelial dysfunction pathway: lower Zeff values may signal more severe endothelial injury, which could further impair cerebral microcirculation and barrier integrity, gradually aggravating CSVD burden over time (42, 48, 49). These pathways are unlikely to act in isolation—rather, they probably overlap and interact, ultimately converging on a common route of cerebral microvascular injury. CSVD burden provides an integrated quantitative measure of chronic cumulative cerebral microvascular injury (50). While our findings suggest a directional association between plaque-related imaging parameters and CSVD burden, the proposed mechanistic interpretations remain speculative and require histopathological confirmation or longitudinal studies to determine causal relationships.

Traditional carotid artery stenosis and plaque thickness measures were not sufficient for improving CSVD risk stratification. By contrast, incorporating Zeff enhanced model performance. Stenosis-based metrics primarily reflect luminal narrowing, whereas Zeff directly quantifies plaque composition, thereby better capturing potentially vulnerable features, including lipid cores, hemorrhagic components, and fibrous tissue. Regression analyses further showed that Zeff was independently associated with CSVD burden, while stenosis parameters were not. These findings suggest that plaque composition assessment may have greater pathophysiological relevance than luminal stenosis assessment. Importantly, intracranial large vessel stenosis lost statistical significance following multivariate adjustment, indicating that CSVD-related brain damage burden is more strongly associated with plaque biological characteristics and systemic vascular pathology rather than anatomical stenosis alone. Subgroup stratification and interaction analysis confirmed consistent associations between Z-Zeff and higher CSVD burden across diverse clinical subgroups, supporting its potential value for classifying high CSVD burden within the studied cohort, while external validation remains required. However, these exploratory findings require external validation before broader application.

This study has several limitations. First, the cross-sectional design with only baseline DLSCT and MRI precludes assessment of longitudinal CSVD progression and causal relationships. Mechanistic interpretations are therefore hypothesis-generating, and a prospective longitudinal cohort with extended follow-up is required to clarify temporal and causal relationships. Second, external validation is lacking because models were developed in a single institution with internal bootstrap validation. Multi-center prospective validation is required to establish generalizability, calibration and discrimination. The skewed CSVD burden score distribution precluded reliable ordinal regression, and finer ordinal strata require larger, more balanced cohorts. Third, findings mainly apply to hospitalized high-risk patients with carotid atherosclerosis undergoing relevant imaging. Their generalizability to asymptomatic community individuals requires validation. Fourth, carotid plaque composition was not histologically confirmed. DLSCT parameters are imaging surrogates and cannot determine specific histopathological constituents, so further histopathological correlation is needed. Finally, manual ROI delineation is susceptible to operator-dependent variability. Automated or semi-automated segmentation will be essential to enhance measurement consistency and reproducibility.

## 5 Conclusion

This study demonstrated that DLSCT-derived Zeff is an independent quantitative imaging parameter associated with CSVD burden. Furthermore, a model integrating DLSCT quantitative parameters and plaque morphological features with clinical variables was developed and evaluated in association with higher CSVD burden. These findings support further investigation of DLSCT-derived quantitative parameters as imaging correlates of CSVD burden in hospitalized patients with carotid atherosclerosis.

## Data Availability

The original contributions presented in the study are included in the article/supplementary material, further inquiries can be directed to the corresponding author.
