# Supplementary material for: Quantitative parameters of carotid plaque based on dual-layer spectral-detector CT for stratifying cerebral small vessel disease
Source: Front Neurol. 2026 Oct 21;17:1911566. doi: 10.3389/fneur.2026.1911566 (PMC13733401; doi:10.3389/fneur.2026.1911566)
Supplement: Supplementary file 1 [file Table_1.docx]

**Quantitative parameters of carotid plaque based on** **dual-layer spectral-detector CT for stratifying cerebral small vessel disease**

**Yuxin Ye^1,3+^, Lin Lin^2+^, Yuxuan Liu^1,3^, Chunmiao Lin^3*^, Yuyun Xu^1,2,3*^**

^1^The Second School of Clinical Medicine, Hangzhou Normal University, Hangzhou, Zhejiang, China

^2^School of Public Health, Hangzhou Medical College, Hangzhou, Zhejiang, China

^3^Center for Rehabilitation Medicine, Department of Radiology, Zhejiang Provincial People's Hospital (Affiliated People's Hospital), Hangzhou Medical College, Hangzhou, Zhejiang, China.

***Correspondence:** Chunmiao Lin, Email: [lcmdb@126.com](mailto:lcmdb@126.com);

Yuyun Xu, Email: [xuyuyun@hmc.edu.cn](mailto:xuyuyun@hmc.edu.cn)

^+^These authors contributed equally to this work.

**Table S1 Inter-observer and intra-observer reproducibility of DLSCT parameters and CSVD burden score assessed by ICC and Bland-Altman analysis**

| **DLSCT parameters** | **Inter-observer ICC (95% CI)** | **Intra-observer ICC (95% CI)** | **Bland-Altman** | **Bland-Altman 95% LoA** |
| --- | --- | --- | --- | --- |
| Zeff | 0.922 (0.872-0.954) | 0.946 (0.908-0.969) | 0.01 | -0.19-0.21 |
| λHU | 0.901 (0.838-0.940) | 0.928 (0.878-0.957) | -0.02 | -0.22-0.19 |
| ED | 0.885 (0.810-0.931) | 0.912 (0.853-0.947) | 0.01 | -0.06-0.07 |
| ID | 0.867 (0.782-0.920) | 0.893 (0.824-0.935) | 0.03 | -0.21-0.27 |
| CSVD burden score | 0.985 (0.972-0.992) | 0.986 (0.974-0.993) | -0.02 | -0.30-0.26 |

*Notes: All DLSCT parameters demonstrated excellent inter- and intra-observer reproducibility with ICC values >0.85. Abbreviations: DLSCT, dual-layer spectral computed tomography; ICC, intraclass correlation coefficient; CI, confidence interval; λHU, spectral curve slope; Zeff, effective atomic number; ED, effective electron density; ID, iodine density.*

**Table S2 MRI acquisition parameters**

| **Sequence** | **Feature** | **GE DISCOVERY MR750 3.0 T** | **SIEMENS Skyra 3.0 T** |
| --- | --- | --- | --- |
| Coil channels |  | 8-channel head coil | 32-channel head coil |
| T1WI | TR (ms) | 1740 | 1500 |
|  | TE (ms) | 27 | 7 |
|  | TI (ms) | 750 | 683 |
|  | slice thickness (mm) | 5.5 | 5 |
| T2WI | TR (ms) | 5752 | 4800 |
|  | TE (ms) | 101 | 95 |
|  | slice thickness (mm) | 5.5 | 5.0 |
| FLAIR | TR (ms) | 8500 | 7400 |
|  | TE (ms) | 120 | 85 |
|  | TI (ms) | 2412 | 2281 |
|  | slice thickness (mm) | 5.5 | 5 |
| DWI | TR (ms) | 3051 | 4700 |
|  | TE (ms) | 63 | 98 |
|  | slice thickness (mm) | 5.5 | 5 |
| SWI | TR (ms) | 37 | 27 |
|  | TE (ms) | 23.1 | 20 |
|  | slice thickness (mm) | 1.6 | 1.5 |

*Abbreviations:* *MRI, magnetic resonance imaging; T1WI, T1-weighted imaging; T2WI, T2-weighted imaging; FLAIR, fluid-attenuated inversion recovery; DWI, diffusion-weighted imaging; SWI, susceptibility-weighted imaging; TR, repetition time; TE, echo time; TI, inversion time.*

**Table S3 Brant test for the proportional odds assumption and convergence of the partial proportional odds model**

| **Model** | **Variable** | **Brant Test χ²** | **df** | ***p*** |
| --- | --- | --- | --- | --- |
| Model 1 | Omnibus | 10.081 | 9 | 0.344 |
| Model 1 | Z-Age | 1.624 | 3 | 0.654 |
| Model 1 | Hypertension | 0.892 | 3 | 0.827 |
| Model 1 | Diabetes | 0.724 | 3 | 0.867 |
| Model 2 | Omnibus | 14.302 | 18 | 0.710 |
| Model 2 | Z-Age | 1.755 | 3 | 0.625 |
| Model 2 | Hypertension | 1.195 | 3 | 0.754 |
| Model 2 | Diabetes | 0.580 | 3 | 0.901 |
| Model 2 | Z-Non-calcified plaque thickness | 3.554 | 3 | 0.314 |
| Model 2 | Carotid lumen stenosis | 1.807 | 6 | 0.937 |
| Model 3 | Omnibus | 19.876 | 12 | 0.069 |
| Model 3 | Z-Age | 1.456 | 3 | 0.693 |
| Model 3 | Hypertension | 0.876 | 3 | 0.831 |
| Model 3 | Diabetes | 0.543 | 3 | 0.909 |
| Model 3 | Z-Zeff | 18.248 | 3 | < 0.001 |
| Model 4 | Omnibus | 6.542 | 21 | 0.999 |
| Model 4 | Z-Age | 0.179 | 3 | 0.914 |
| Model 4 | Hypertension | 1.385 | 3 | 0.709 |
| Model 4 | Diabetes | 0.036 | 3 | 0.982 |
| Model 4 | Z-Non-calcified plaque thickness | 9.837 | 3 | 0.020 |
| Model 4 | Carotid lumen stenosis | 4.561 | 6 | 0.102 |
| Model 4 | Z-Zeff | 18.247 | 3 | < 0.001 |

*Note: Brant test P < 0.05 indicates violation of the proportional odds assumption. Partial proportional odds models were explored after violations of the proportional odds assumption were identified but did not converge, likely because of sparse observations in the highest CSVD score categories, including 35 participants with a score of 3 and 6 with a score of 4. Therefore, the proportional odds ordinal logistic regression model was not considered appropriate for the primary analysis, and CSVD burden was dichotomized as low (<2) or high (≥2). Abbreviations: CSVD, cerebral small vessel disease; Zeff, effective atomic number; Z-Zeff, Z-score-standardized effective atomic number.*

**Table S4 Sensitivity analyses using alternative Zeff measurements for the association with high CSVD burden**

| **Zeff measurement** | **OR (95% CI)** | ***p*** | **Optimism-corrected AUC (95% CI)** |
| --- | --- | --- | --- |
| Zeff at the plaque with the most severe stenosis | 0.386 (0.256–0.581) | <0.001 | 0.759 (0.708–0.835) |
| Lowest Zeff across bilateral carotid plaques | 0.210 (0.120–0.370) | <0.001 | 0.740 (0.690–0.790) |
| Mean Zeff across all measurable carotid plaques | 0.230 (0.140–0.380) | <0.001 | 0.745 (0.695–0.795) |

*Note: ORs are expressed per 1-SD increase in the respective Zeff measurement. All models were adjusted for Z-age, hypertension, diabetes, Z-non-calcified plaque thickness, and carotid lumen stenosis. AUC values are optimism-corrected using 1,000 bootstrap resamples. Abbreviations: AUC, area under the receiver operating characteristic curve; OR, odds ratio; SD, standard deviation; CI, confidence interval; Zeff, effective atomic number.*

**Table S5 Statistical test for model comparison**

| **Model comparison** | **ΔAUC (95% CI)** | **DeLong's p-value** | **likelihood ratio test χ² (p-value)** | **ΔBrier score** | **Corrected Calibration Slope** |
| --- | --- | --- | --- | --- | --- |
| Model2 vs Model1 | 0.015 (-0.017-0.047) | 0.768 | 3.66 (0.300) | -0.003 | M1: 0.794 / M2: 0.916 |
| Model3 vs Model1 | 0.124 (0.057-0.191) | < 0.001 | 29.82 (< 0.001) | -0.032 | M1: 0.794 / M3: 0.860 |
| Model3 vs Model2 | 0.109 (0.044-0.174) | < 0.001 | *—* | -0.029 | M2: 0.916 / M3: 0.860 |
| Model4 vs Model2 | 0.120 (0.058–0.183) | < 0.001 | 27.63 (< 0.001) | -0.034 | M2: 0.916 / M4: 0.933 |
| Model4 vs Model3 | 0.011 (−0.002–0.025) | 0.644 | 1.47 (0.688) | -0.005 | M3: 0.860 / M4: 0.933 |
| Model4 vs Model1 | 0.135 (0.067–0.203) | < 0.001 | 31.29 (< 0.001) | -0.037 | M1: 0.794 / M4: 0.933 |

*Note：—: Indicates that the method is not applicable (since the Model 3 and Model 2 do not have a nested relationship, a likelihood ratio test is not performed). Model 1 = age+hypertension+diabetes; Model 2 = Model 1 +non-calcified plaque thickness+carotid lumen stenosis; Model 3 = Model 1+Z-Zeff; Mode 4 = age+hypertension+diabetes+non-calcified plaque thickness+carotid lumen stenosis+Z-Zeff. Abbreviations: AUC, area under the receiver operating characteristic curve; ΔAUC, incremental AUC; LRT, Likelihood Ratio Test; CI, 95% confidence interval; ΔBrier Score, difference in Brier scores between models; Corrected Calibration Slope, Bootstrap-optimized calibration slope; All statistical metrics have been internally validated using 1,000 bootstrap samples.*

**Table S6 Stratified analysis of the association between Z-Zeff and high CSVD burden by clinical characteristics**

| Stratification variable | Subgroup | OR (95% CI) | *P* for interaction |
| --- | --- | --- | --- |
| Previous ischemic stroke with old small infarct (maximum diameter ≤ 15 mm) or TIA | Yes | 0.34 (0.20–0.58) | 0.682 |
|  | No | 0.29 (0.16–0.52) |  |
| Acute small infarction | Yes | 0.26 (0.11–0.61) | 0.547 |
|  | No | 0.32 (0.20–0.51) |  |
| Symptomatic carotid artery disease | Yes | 0.22 (0.08–0.60) | 0.891 |
|  | No | 0.31 (0.19–0.50) |  |

*Notes: ORs were adjusted for age, hypertension, diabetes, non-calcified plaque thickness, and carotid lumen stenosis (Model 4 covariates), except for the stratification variable itself. Z-Zeff was Z-score standardized; the OR represents the change in odds of high CSVD burden per 1-SD increase in Z-Zeff. p for interaction was calculated by adding a Z-Zeff × subgroup interaction term to the fully adjusted model. Abbreviations: CSVD, cerebral small vessel disease; TIA, transient ischemic attack; Zeff, effective atomic number; OR, odds ratio; CI, confidence interval; SD, standard deviation.*

**Table S7 Sensitivity analyses for the association between Z-Zeff and high CSVD burden after excluding patients receiving antiplatelet therapy**

| **Analysis** | **Model** | **Z-Zeff OR (95% CI)** | ***p*** | **Optimism-corrected AUC (95% CI)** | **Optimism-corrected calibration slope** | **Optimism-corrected Brier score** |
| --- | --- | --- | --- | --- | --- | --- |
| Primary analysis | Model 3 | 0.387 (0.262–0.572) | <0.001 | 0.748 (0.712–0.837) | 0.860 | 0.204 |
| Primary analysis | Model 4 | 0.386 (0.256–0.581) | <0.001 | 0.759 (0.708–0.835) | 0.933 | 0.199 |
| Sensitivity analysis | Model 3 | 0.420 (0.230–0.780) | 0.006 | 0.720 (0.650–0.790) | 0.850 | 0.210 |
| Sensitivity analysis | Model 4 | 0.440 (0.240–0.810) | 0.008 | 0.730 (0.660–0.800) | 0.880 | 0.200 |

*Note: Z-Zeff indicates Z-score-standardized effective atomic number. OR represents the odds ratio per 1-SD increase in Z-Zeff. Model 3 = Model 1+Z-Zeff; Mode 4 = age+hypertension+diabetes+non-calcified plaque thickness+carotid lumen stenosis+Z-Zeff. Abbreviations: CSVD, cerebral small vessel disease; Zeff, effective atomic number; OR, odds ratio; AUC, area under the curve;CI, confidence interval; SD, standard deviation.*
